# Supplementary material for: Control of bacterial quorum threshold for metabolic homeostasis and cooperativity
Source: Microbiol Spectr. 2023 Dec 12;12(1):e03353-23. doi: 10.1128/spectrum.03353-23 (PMC10783058; doi:10.1128/spectrum.03353-23)
Supplement: Supplemental Figures — Figures S1 to S7. [file spectrum.03353-23-s0001.pdf]

**SUPPLEMENTAL MATERIAL**

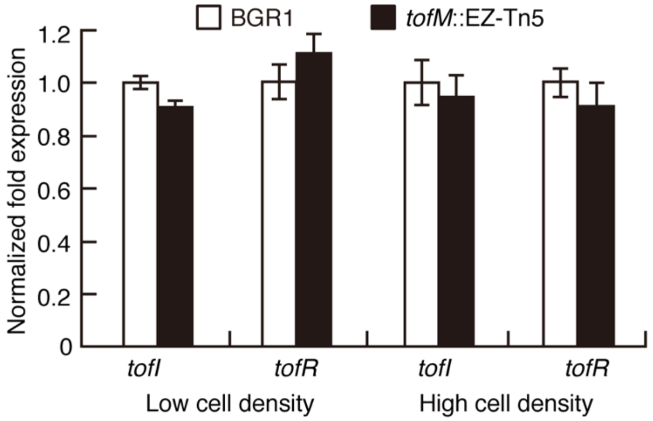


**FIG S1** Comparison of normalized fold expression of *tofI* and *tofR* genes in the wild-type BGR1 and the *tofM* mutant. Reverse transcription quantitative PCR results demonstrated that there were no significant differences in the transcription levels of both the *tofI* and *tofR* genes between the *tofM* mutant and the wild-type BGR1, either at approximately 3 x $10^8$ CFU/mL (low cell density, LCD) or 2.4 x $10^9$ CFU/mL (high cell density, HCD). The relative expression levels of the *tofI* and *tofR* genes are calculated by using values of the BGR1 strain at LCD and HCD, after normalization to the expression levels of the 16S rRNA gene. Normalized fold expression levels of the *tofI* and *tofR* genes in the BGR1 and *tofM* mutant are represented by white bars and black bars, respectively. The data represent the mean ± standard deviation of triplicate experiments. Statistical analysis was performed using one-way analysis of variance (ANOVA) (LCD; $F_{tofI}$ (1,4) = [1146.848], $p_{tofI}$ = 0.001, $F_{tofR}$ (1,4) = [292.368], $p_{tofR}$ = 0.001, HDC; $F_{tofI}$ (1,4) = [0.084], $p_{tofI}$ = 0.786, $F_{tofR}$ (1,4) = [0.193], $p_{tofR}$ = 0.683).

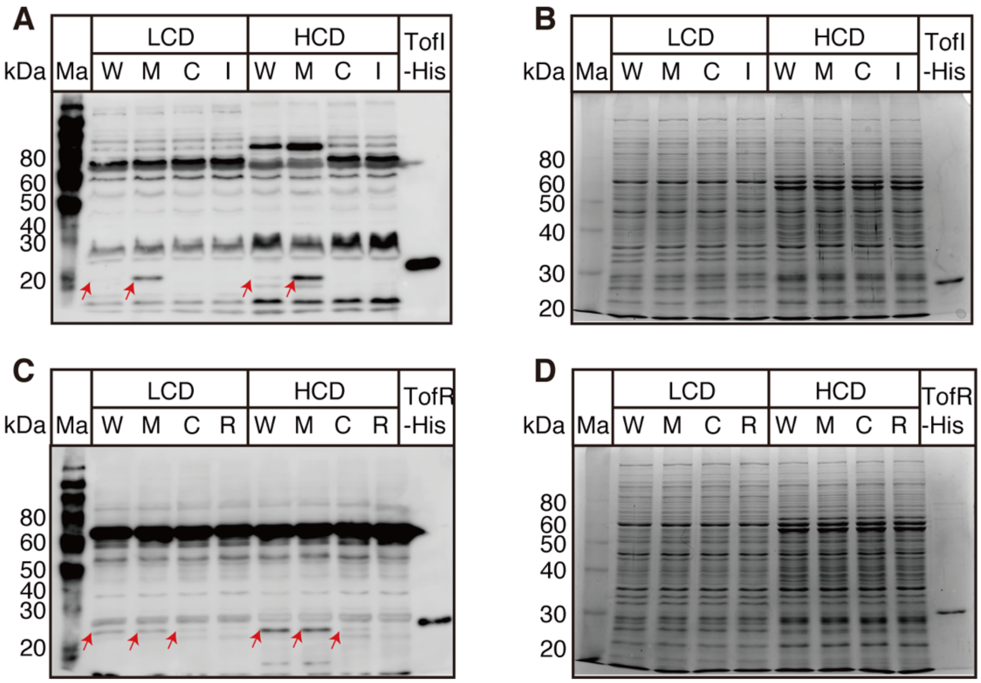


**FIG S2** High levels of the QS signal synthase TofI enzyme were produced by the *tofM* mutant. (A) The amount of TofI in each strain was determined via Western blot analysis using an anti-TofI antibody. (B) The image of the SDS-PAGE gel stained with Coomassie brilliant blue R-250 (Sigma) shows that samples were loaded equally in all lanes. (C) Western blot using an anti-TofR antibody indicated similar levels of TofR in both the wild type and *tofM* mutant. (D) The image of the SDS-PAGE gel after staining with Coomassie brilliant blue R-250 (Sigma) was photographed using ChemiDoc MP imaging system (Bio-Rad). Ma; molecular marker, LCD; low cell density (3.0 × $10^8$ CFU/mL), HCD; high cell density (2.4 × $10^9$ CFU/mL), W; wild type, M; *tofM* mutant, C; complementation strain of the *tofM* mutant, I; *tofI* mutant, R; *tofR* mutant. All data are representative of triplicate experiments. Red arrows indicate the corresponding bands of TofI or TofR from each result.

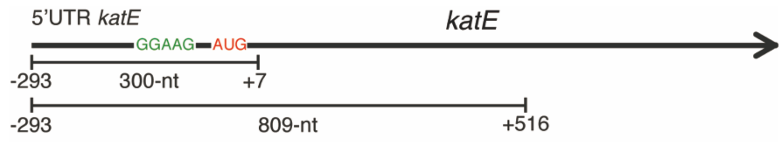


**FIG S3** Map of *katE* ORF. Map is showing the putative promoter region including ribosome binding site (green). The transcription start codon AUG is highlighted in red.

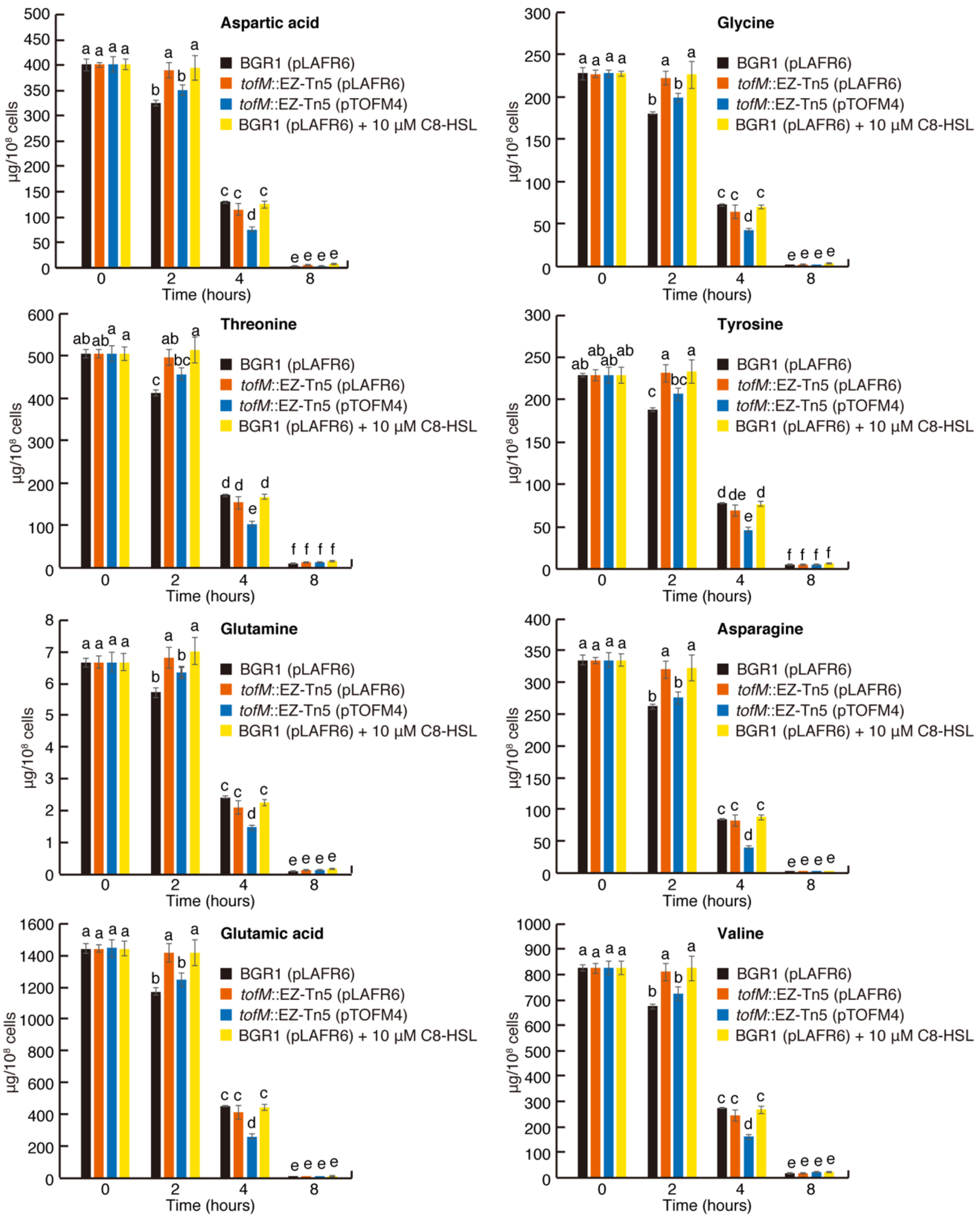
Aspartic acid
BGR1 (pLAFR6)
tofM::EZ-Tn5 (pLAFR6)
tofM::EZ-Tn5 (pTOFM4)
BGR1 (pLAFR6) + 10 µM C8-HSL
µg/10⁸ cells
Time (hours)
Glycine
BGR1 (pLAFR6)
tofM::EZ-Tn5 (pLAFR6)
tofM::EZ-Tn5 (pTOFM4)
BGR1 (pLAFR6) + 10 µM C8-HSL
µg/10⁸ cells
Time (hours)
Threonine
BGR1 (pLAFR6)
tofM::EZ-Tn5 (pLAFR6)
tofM::EZ-Tn5 (pTOFM4)
BGR1 (pLAFR6) + 10 µM C8-HSL
µg/10⁸ cells
Time (hours)
Tyrosine
BGR1 (pLAFR6)
tofM::EZ-Tn5 (pLAFR6)
tofM::EZ-Tn5 (pTOFM4)
BGR1 (pLAFR6) + 10 µM C8-HSL
µg/10⁸ cells
Time (hours)
Glutamine
BGR1 (pLAFR6)
tofM::EZ-Tn5 (pLAFR6)
tofM::EZ-Tn5 (pTOFM4)
BGR1 (pLAFR6) + 10 µM C8-HSL
µg/10⁸ cells
Time (hours)
Asparagine
BGR1 (pLAFR6)
tofM::EZ-Tn5 (pLAFR6)
tofM::EZ-Tn5 (pTOFM4)
BGR1 (pLAFR6) + 10 µM C8-HSL
µg/10⁸ cells
Time (hours)
Glutamic acid
BGR1 (pLAFR6)
tofM::EZ-Tn5 (pLAFR6)
tofM::EZ-Tn5 (pTOFM4)
BGR1 (pLAFR6) + 10 µM C8-HSL
µg/10⁸ cells
Time (hours)
Valine
BGR1 (pLAFR6)
tofM::EZ-Tn5 (pLAFR6)
tofM::EZ-Tn5 (pTOFM4)
BGR1 (pLAFR6) + 10 µM C8-HSL
µg/10⁸ cells
Time (hours)
0
2
4
8

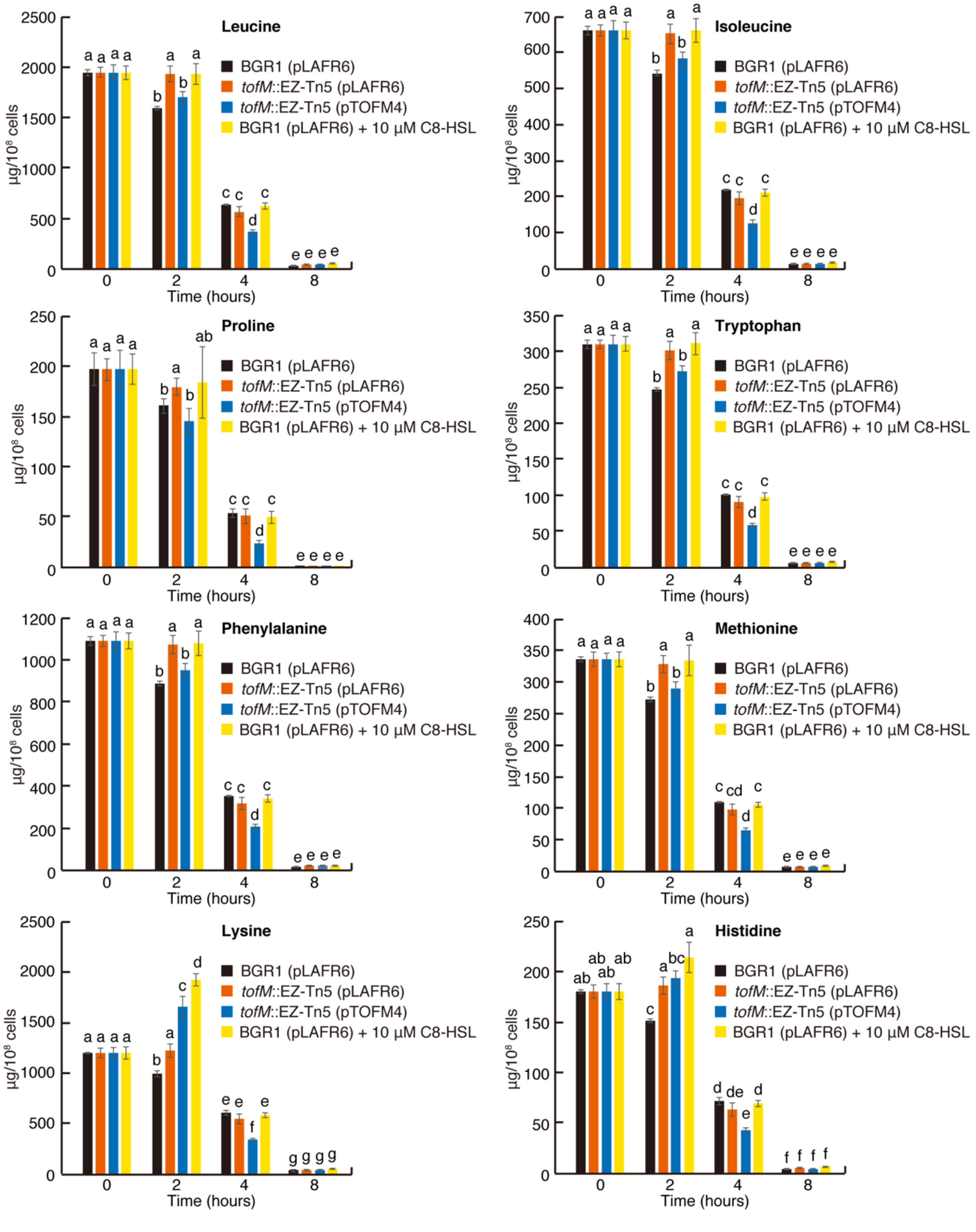


**FIG S4** Metabolic slowing in the *tofM* mutant at the early growth stage. The levels of aspartic acid, glycine, threonine, tyrosine, glutamine, asparagine, glutamic acid, valine, leucine, isoleucine, proline, tryptophan, phenylalanine, methionine, lysine, and histidine in the culture supernatants of the wild type, *tofM* mutant, complementation strain of the *tofM* mutant, and the

wild type supplemented with 10 μM C8-HSL were determined by high-performance liquid chromatography (HPLC, Thermo Dionex) at designated time points. Amino acids levels were normalized through division by bacterial density (CFU/mL) and expressed as μg/$10^8$ cells. The data shows the mean ± standard deviation of triplicate experiments. The letters (a, b, c, d, e, f, and g) above each mean represent significant differences based on a one-way ANOVA, followed by Tukey's post-hoc analysis. A separate ANOVA was conducted for each time point in the figures, rather than a single ANOVA covering all measurements at all time points. A value of $p < 0.05$ represented significant differences among strains.

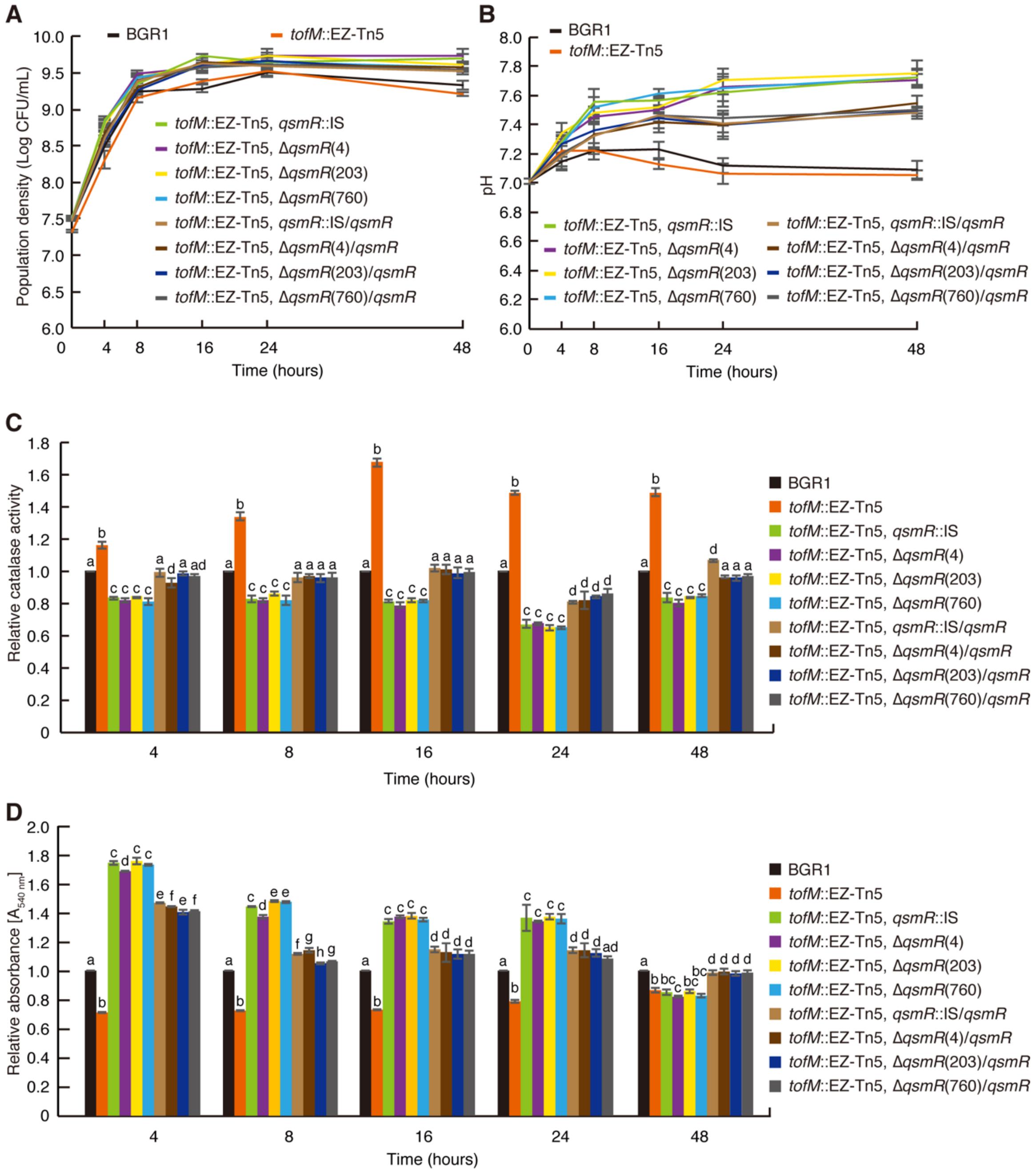


**FIG S5** Growth, pH, catalase activity and metabolic activity of spontaneous *qsmR* mutants. (A) The growth of the wild-type BGR1, *tofM* mutant, four types of *tofM*/*qsmR* mutant, four types of *qsmR* complementation strains of *tofM*/*qsmR* mutant in LB broth was monitored over 48 h of closed batch culturing. The population density was measured as colony forming units (CFU) per mL, and the data are the means of three biological replicates ± standard deviation. (B) The extracellular pH, (C) catalase activity, and (D) metabolic activity were measured at designated

time points in each strain during growth in LB broth. The relative catalase activity and relative metabolic activity were calculated by dividing the levels measured in each strain by that of the wild type. The data represent the means of three biological replicates ± standard deviation. The letters (a, b, c, d, e, f, g, and h) above each mean represent significant differences based on a one-way ANOVA, followed by Tukey's post-hoc analysis. A separate ANOVA was conducted for each time point in the figures, rather than a single ANOVA covering all measurements at all time points. A value of $p < 0.05$ represented significant differences among strains.

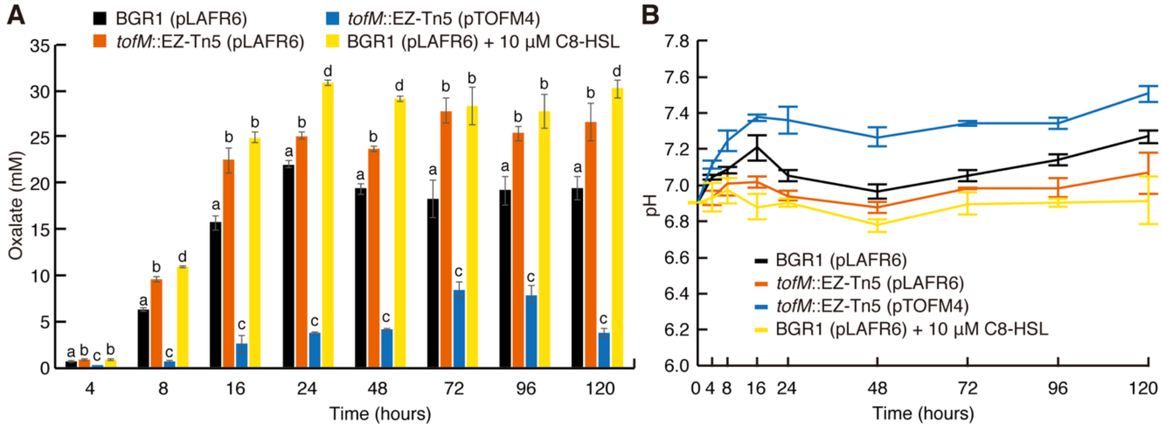


**FIG S6** The levels of oxalate and pH in each strain. The *tofM* mutant or the BGR1 supplemented with 10 μM C8-HSL showed higher levels of oxalate synthesis compared to the wild-type BGR1, but there was no significant difference in environmental pH between the strains. (A) Oxalate and (B) pH were monitored at designated time points in each strain during growth in LB broth. The data represent the means of three biological replicates ± standard deviation. The letters (a, b, c, and d) above each mean represent significant differences based on a one-way ANOVA, followed by Tukey's post-hoc analysis. A separate ANOVA was conducted for each time point in the figures, rather than a single ANOVA covering all measurements at all time points. A value of $p < 0.05$ represented significant differences among strains.

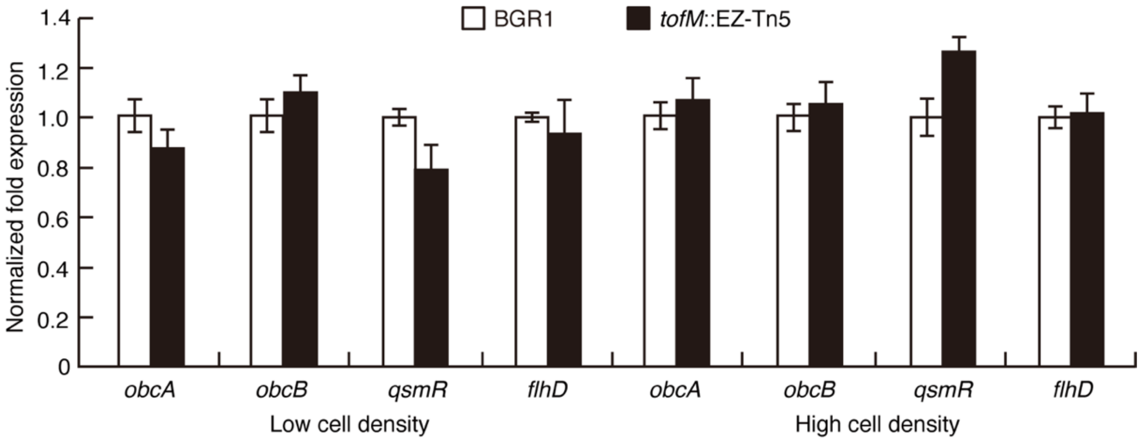


**FIG S7** Comparison of normalized fold expression of *obcA, obcB, qsmR* and *flhD* genes in the wild-type BGR1 and the *tofM* mutant. Reverse transcription quantitative PCR results demonstrated that there were no significant differences in the transcription levels of the *obcA*, *obcB*, *qsmR*, and *flhD* genes between the *tofM* mutant and the wild-type BGR1, either at approximately $3.0 \times 10^8$ CFU/mL (low cell density, LCD) or $2.4 \times 10^9$ CFU/mL (high cell density, HCD). The relative expression levels of the *obcA*, *obcB*, *qsmR*, and *flhD* genes are calculated by using values of the BGR1 strain at LCD and HCD, after normalization to the expression levels of the 16S rRNA gene. Normalized fold expression levels of the *obcA*, *obcB*, *qsmR*, and *flhD* genes in the BGR1 and *tofM* mutant are represented by white bars and black bars, respectively. The data represent the mean ± standard deviation of triplicate experiments. Statistical analysis was performed using one-way analysis of variance (ANOVA) (LCD; $F_{obcA}$ (1,4) = [2.387], $p_{obcA}$ = 0.197, $F_{obcB}$ (1,4) = [49.330] $p_{obcB}$ = 0.002, $F_{qsmR}$ (1,4) = [0.479], $p_{qsmR}$ = 0.527, $F_{flhD}$ (1,4) = [47.473], $p_{flhD}$ = 0.002, HDC; $F_{obcA}$ (1,4) = [0.557], $p_{obcA}$ = 0.497, $F_{obcB}$ (1,4) = [0.510] $p_{obcB}$ = 0.515, $F_{qsmR}$ (1,4) = [11.727], $p_{qsmR}$ = 0.027, $F_{flhD}$ (1,4) = [1.233], $p_{flhD}$ = 0.329).
