# Supplementary material for: Control of bacterial quorum threshold for metabolic homeostasis and cooperativity
Source: Microbiol Spectr. 2023 Dec 12;12(1):e03353-23. doi: 10.1128/spectrum.03353-23 (PMC10783058; doi:10.1128/spectrum.03353-23)
Supplement: Supplemental Tables-references — Tables S1 to S3. [file spectrum.03353-23-s0002.pdf]

TABLE S1 Homologs of *Burkholderia glumae* TofM in *Pseudomonas aeruginosa* PAO1 and *P. fuscovaginae* UPB0736

Strain	Description	Accession number	Score (bits)	Query cover (%)	e-value	Identities (%)
P. fuscovaginae UPB0736	RsaM	CBI67624.1	45.8	68	1e-11	30.39
P. aeruginosa PAO1	RsmA	AAG04294.1	17.3	18	0.019	32.14

TABLE S2 Strains and plasmids used in this study

Strains	Characteristics	Source or Reference
B. glumae		
BGR1	Wild type, Rif^R	1
BTOFM	BGR1 *tofM*::EZ-Tn5, Tp^R	This study
BTOFMC	BGR1 *tofM*::EZ-Tn5/*tofM*	This study
BGS2	BGR1 *tofI*::Ω, Sp^R	1
BGS3	BGR1 *tofR*/*I*/*M*::Ω, Sp^R	This study
BTOFM_Cr6	Spontaneous mutant from batch cultures of *tofM*::EZ-Tn*5*, IS1418 insertion in *qsmR* gene	This study
BTOFM_Cr1	Spontaneous mutant from batch cultures of *tofM*::EZ-Tn*5*, 4-bp deletion in *qsmR* gene	This study
BTOFM_Ly1	Spontaneous mutant from batch cultures of *tofM*::EZ-Tn*5*, 203-bp deletion in *qsmR* gene	This study
BTOFM_Cr3	Spontaneous mutant from batch cultures of *tofM*::EZ-Tn*5*, 760-bp deletion in *qsmR* gene	This study
BTOFM_Cr6C	Complementation strain of spontaneous mutant from batch cultures of *tofM*::EZ-Tn*5*, IS1418 insertion in *qsmR* gene	This study
BTOFM_Cr1C	Complementation strain of spontaneous mutant from batch cultures of *tofM*::EZ-Tn*5*, 4-bp deletion in *qsmR* gene	This study
BTOFM_Ly1C	Complementation strain of spontaneous mutant from batch cultures of *tofM*::EZ-Tn*5*, 203-bp deletion in *qsmR* gene	This study
BTOFM_Cr3C	Complementation strain of spontaneous mutant from batch cultures of *tofM*::EZ-Tn*5*, 760-bp deletion in *qsmR* gene	This study

Escherichia coli DH5α	F[-] Φ80d*lacZ*Δ*M15* Δ(*lacZYA-argF*)*U169 endA1 recA1 hsdR17* ($r_K^- m_K^+$) *deoR thi-1 supE44 λ-gyrA96 relA*	Gibco BRL, Waltham, MA, USA
BL21(DE3)	F[-]*ompT hsdS*$_B$($r_B^- m_B^-$)*gal dcm* (DE3)	Novagen
Chromobacterium violaceum CV026	Autoinducer indicator strain	3
Plasmids		
pBluescript II SK(+)	Cloning vehicle; phagemid, pUC derivative, Amp^R	Stratagene, Santa Clara, CA, USA
pLAFR3	Tra$^-$, Mob$^+$, RK2 replicon, Tet^R	4
pLAFR6	As pLAFR3 but without *lacZα*, contains multilinker of pUC18 flanked by synthetic *trp* terminators, Tet^R	5
pTOFM1	528-bp PCR fragment including *tofM* gene from strain BGR1 cloned into pBluescript II SK(+)	This study
pTOFM2	528-bp *N*deI-*H*indIII DNA fragment from pTOFM1 clone into pET-21b	This study
pTOFM3	528-bp PCR fragment including native promoter and *tofM* gene from strain BGR1 cloned into pBluescript II SK(+)	This study
pTOFM4	528-bp *Eco*RI-*B*amHI DNA fragment from pTOFM3 clone into pLAFR6	This study
pTOFI6	0.697-kb fragment including native promoter and *tofI* gene cloned into pLAFR6	2
pBGA18	25.3-kb DNA fragment including *tofM* gene from strain BGR1 cloned into pLAFR3	1
pBGA18::EZ-Tn5	EZ-Tn5 (Tp^R) inserted into pBGA18	This study
pBGA38R	7.9-kb *B*amHI-*H*indII fragment from pBGA37R cloned into pLAFR3	1

pReuscue-mini-Tn5_qsmR	pUTKm *oriV* including native promoter and *qsmR* gene but without polylinker, enable rescue of the Tn5 tagging region	6
pRK2013	Helper plasmid, Tra^{+}, ColE1 replicon, Km^{R}	7

TABLE S3 Primers used in this study

Primer name	Sequence (5′→3′)
EcoRI_ptofM-F	GGGAATTCCAATCTCTCCAACAGCTTCCAC
BamHI_stop_His_tofM-R	GGGATCCTCAGTGGTGATGATGGTGATGGTGCGACCAGCGTTTGTCG
NdeI_tofM-F	CCCATATGACTCCGCCCCTGCTTCAT
tofM_XhoI-R	CCCTCGAGGTGCGACCAGCGTTTGTC
RT_tofR-F	TGCCTACCACCAGTTCAATAC
RT_tofR-R	GTGTCGAAGATCTCGACCAAG
RT_tofI-F	TTGGGACGCGATGCGAATG
RT_tofI-R	GAAGCGCGACAGCTCCCAGA
RT_obcA-F	AATCGCCGGCATTATTGAA
RT_obcA-R	TCGAGCGAATGGTTTCCTCG
RT_obcB-F	CAGGACGGTGCGGTGCTGCATA
RT_obcB-R	TGAATGCCGACCAGGCTGTAGT
RT_qsmR-F	CGCGAGCTGACAGAATTGA
RT_qsmR-R	ACCGAGGCGGTCAGTACGAA
RT_flhD-F	CGAGATCAAGGAAGTTAAC
RT_flhD-R	AGCACGTCCGCAATCTCTTC
PT7tofI-F	TTTCTGCAGTAATACGACTCACTATAGGGAATCCGAGATATCGCGCCGACCCT
PtofI-R	ATCGTCGCGATCGTACTGATC
tofI-17nt_tail-R	AAAAAAAACCCCCCCCCGGCCGCTTCGGGTTGCGACG
PT7katE-F	TTTCTGCAGTAATACGACTCACTATAGGGACTCGCGCCGCTCGTCGAAGCGCAT
PkatE-R	TGGCCATGCACTTCCTCCACT
MkatE-R	GATCAGGTCCGGGAACTTGAT
ptofI-F	CTTCGCGACGCCTCGACTTTAATCC
tofI-R	TCAGGCCGCTTCGGGTTGCGA
tofR-F	GCGCGGGAATGTATCAAGCCCGTGTGCTT
tofR-R	CTACGGCGCCTGGATCAGCCCCAT
qsmR-F	GAAATGAGGGAGACCAGTCTGTCTATT
qsmR-R	GTTTGCGGTTCCGGGTTATTCATGTTCGATC
BamHI-obcA-F	AGCTAGCGGATCCGGACGGATGGGGTCCGATTTTCGG
obcB-EcoRI-R	CATGCATGAATTCTCACCGCGTCACGCGTACCAGCT

REFERENCES

1. **Kim J, Kim JG, Kang Y, Jang JY, Jog GJ, Lim JY, Kim S, Suga H, Nagamatsu T, Hwang I.** 2004. Quorum sensing and the LysR-type transcriptional activator ToxR regulate toxoflavin biosynthesis and transport in *Burkholderia glumae*. Mol Microbiol **54**: 921–934.
2. **Goo E, Hwang I.** 2021. Essential roles of Lon protease in the morpho-physiological traits of the rice pathogen *Burkholderia glumae*. PLoS One **16**: e0257257.
3. **McClean KH, Winson MK, Fish L, Taylor A, Chhabra SR, Camara M, Daykin M, Lamb JH, Swift S, Bycroft BW, Stewart GSAB, Williams P.** 1997. Quorum sensing and *Chromobacterium violaceum*: exploitation of violacein production and inhibition for the detection of *N*-acyl homoserine lactones. Microbiol **143**: 3703-3711.
4. **Staskawicz B, Dahlbeck D, Keen N, Napoli C.** 1987. Molecular characterization of cloned avirulence genes from race 0 and race 1 of *Pseudomonas syringae* pv. *glycinea*. J Bacteriol **169**: 5789-5794.
5. **Huynh TV, Dahlbeck D, Staskawicz BJ.** 1989. Bacterial blight of soybean: regulation of a pathogen gene determining host cultivar specificity. Science **245:** 1374– 1377.
6. **Kang Y, Hwang I.** 2018. Glutamate uptake is important for osmoregulation and survival in the rice pathogen *Burkholderia glumae*. PLoS One **13**: e0190431.
7. **Figurski DH, Helinski DR.** 1979. Replication of origin-containing derivative of plasmid RK2 dependent on a plasmid function provided in *trans*. Proc Natl Acad Sci USA **76**: 1648-1652.
